# Bad Handwriting

**Published:** 1882-03

**Authors:** 


### BAD HANDWRITING.

There are here and there human beings who are by nature incapable of writing a good hand, just as there are others who cannot draw a straight line or a true circle, or even recognize one. But the ugly manuscript of the clumsy-fisted struggler after form is usually very clear. Haste, uneasiness, excessive work, nervous pre-occupation—these are the chief causes of obscure handwriting with most of us. But when a man's manuscript has once made for itself a fixed character of its own, neither printers nor expert copyists would like it to come round to tame simplicity and correctness. It would be, in another way, the case of the lover with a squint, who ruined his suit by going to the oculist and getting his eyes put straight; the lady could no longer meet his eye in the old, affectionate way, and she dismissed him. Still, there are faults of handwriting which are inexcusable in themselves, and which neither compositor nor copyist can possibly like to see. One of the worst of these is lax practice in putting the strokes to such letters as *m* and *n*. There is no harm in cutting down certain syllables, such as *ment* and *ing*, to mere lines or twirls, but where an attempt is made to express the characters, the number of strokes ought to be uniform. Another practical observation is that *flurried* handwriting gains no time for the writer. A downright lazy scrawl is another matter, and so is that kind of bad writing in which we can see in the badness egotistic self-assertion or disregard of the eyes and wits of others. It may be laid down that there is much egotism (associated, it may be, with much kindness) in the man who writes a bad hand, *which never strives to pick itself up.* But, of course, the rule must be applied with greater or less stringency, according to the amount of work that presses upon the producer of the manuscript, his health, his preoccupation and the activity of his self-consciousness.

The "personal equation" in these matters is quite a curious little study. Mr. Cox, of Nottingham, in his manly and everyway beautiful preface of Mr. Lynch's posthumous "Services," tells us that, in spite of what the preacher used to suffer from the *peine forte et dure* of his malady, and the interruptions it brought, the whole service (sermon and prayers) was sometimes written in a single day. "In copying them out for the press," says Mr. Cox, "I have found that each of them furnishes, in the mere penmanship, a long and hard day's work for a man in good health." Well, supposing the service pretty well composed beforehand, the preacher might, of course, write it out more easily than any one could copy it, unless the latter were a penman practiced in swift work. Such a copyist (not a mere law stationer's man) could do one of these services in five hours; some could do it in four, and the copy would be perfectly fair and clear. Still, the majority of copyists think they have done well when they have got through fifteen folios an hour (a folio is seventy-two words), and at that rate one of these services would take about seven hours to write out. Charles Dickens would certainly have found four hours enough, and we have known clever reporters who were fully up to that, the copy being in every way clear and good. Now, it would take a great deal of over-work, worry and confusion to break down into illegibility a handwriting which was capable of that. It is, in good part, a question of physical energy, and something depends upon the question whether the penman man works more from the wrist or more from the shoulder. However, in course of time, under the pressure of hard work, every handwriting will come to show scars. Often the hand of a strong, broad-shouldered man will break down before that of his slighter brother. So long as the scars are honorable scars, got in a fair fight with the difficulties of the bureau, the study, or the editor's room, the handwriting will remain legible to all but dull or inattentive eyes, because the characteristic portions of the words will be retained by the penman.—*Spectator.*

